# Integrated and responsive implementation support strategies: A qualitative analysis of implementation plans to advance systemic social and emotional learning

**DOI:** 10.1186/s43058-025-00830-w

**Published:** 2025-12-30

**Authors:** Sophia H. J. Hwang, Alagia J. Cirolia, Esmeralda M. Michel, CalHOPE Research Committee, Valerie B. Shapiro

**Affiliations:** 1 Department of Psychology, University of Maryland, Baltimore County, Baltimore, MD USA; 2 School of Social Welfare, University of California, Berkeley, 120 Haviland Hall, Berkeley, CA 94720-7400 USA

**Keywords:** Social and emotional learning (SEL), Implementation, Implementation support, Support system, Schools

## Abstract

**Background:**

Guided by the Interactive Systems Framework and the Evidence-Based System for Implementation Support, the current study examines how County Offices of Education can advance systemic SEL in districts and schools to bolster the well-being and mental health of educators and students.

**Method:**

Through the directed qualitative content analysis of plans drafted by County Leaders, we examine how counties envisioned providing implementation support and provide definitions, examples, and frequencies of five commonly used implementation support strategies in real-world practice.

**Results:**

Tools, trainings, and technical assistance were mentioned in the majority of plans, incentives in approximately half the plans, and feedback loops were referenced in only 27% of the plans. We found that implementation support strategies were innovatively combined to build the capacity of local practitioners in integrated and responsive ways. We also present two new dimensions to conceptualize implementation support and advance theory-building in the field. The first dimension is *implementation support strategy orientation*, or the degree to which strategies are responsive to local preferences, resulting in a spectrum of top-down to bottom-up approaches. Second, *implementation support strategy facilitation,* describes the degree to which support strategies range in their level of requisite preparation.

**Conclusions:**

Ultimately, our findings aim to inform implementation planning, advance and refine current frameworks, and narrow the bidirectional gap between research and practice.

Contributions to the literature

- Social and emotional learning (SEL) interventions only work when implemented well. This study focuses on county-level implementation support to facilitate SEL implementation in districts and schools.
- We provide examples of implementation support strategies in real-world settings to highlight how they are used alone and in combination.
- We elaborate upon existing implementation science frameworks by contributing two new conceptualizations: implementation support strategy orientation (how strategies are responsive to local preferences) and implementation support strategy facilitation (how support strategies vary in their level of requisite preparation) to demonstrate the degree to which implementation support can address specific contextual needs.

## Background

Social and emotional learning (SEL) is the process of acquiring intrapersonal and interpersonal capacities, competencies, and skills that help young people experience positive development and academic success [1]. These SEL capacities are cultivated via practices (e.g., teachers facilitating morning meetings), programs (e.g., manualized curricula about navigating conflict), and policies (e.g., legislation to establish restorative practices in schools) [1]. Meta-analytic evidence demonstrates that school-based SEL programs improve academic achievement, behaviors, and attitudes [2, 3]. However, SEL interventions must be implemented well to achieve these positive outcomes [4]. Implementation success depends on effective implementation strategies (e.g., training, technical assistance) [5], which, when aligned with the local context, are a key driver of positive youth outcomes [4, 6].

Relative to how much is known about SEL implementation in classrooms (e.g., [7]), there has been less investigation on the provision of implementation support from the broader educational system. The majority of studies focus on educators delivering SEL practices in classrooms (e.g., [8]); this work provides important solutions to the “last mile problem” in implementation science, which refers to the challenges during the final stages of implementation that prevent interventions from positively impacting their target [9]. However, successful classroom-based implementation is difficult to achieve without adequate, sustainable, and built-in support from within the school (e.g., via leadership support: [10]), and the larger educational system (e.g., districts). For example, although training (e.g., professional development) is the most common form of SEL implementation support, it is both necessary and insufficient for building sustainable, high-quality implementation at scale [11]. More research is needed about coordinated efforts beyond the school, such as at the county or state level, which can provide comprehensive support for school-based SEL implementation. Thus, there is an opportunity to look “upstream” and better understand these implementation support systems improving SEL delivery at scale.

To address this gap, the current paper examines how implementation support strategies^1^ from above the school-level [5, 6] can aid SEL implementation in schools. Scholars have articulated a need to understand what implementation supports – which risk being abstract and theoretical – look like in on-the-ground practices [12]. The present study advances our understanding of the role that County Offices of Education (COEs)^2^ can play in providing implementation support to local educators. Through the qualitative analysis of implementation support plans, we shed light on the range of implementation support strategies envisioned by COE leaders throughout California to advance systemic SEL. We provide examples of these strategies to describe how these efforts manifest in current practice, including how strategies are innovatively combined. We also present a novel conceptualization of how implementation support strategies vary along two dimensions, *implementation support strategy orientation* and *implementation support strategy facilitation*, to capture how support strategies reflect responsiveness and capacity. Taken together, these findings aim to guide implementation planning, contribute to theory, and narrow the research-practice gap.

## The importance and nature of implementation support in social and emotional learning

Evidence-based SEL programs yield positive student outcomes [2] when they are well-implemented, often by teachers in the classroom (e.g., PATHS; [13, 14]). A *systemic* approach to SEL engages youth and adult perspectives through coordinating SEL across all levels of the education system (e.g., schools, districts, and states) [15] to improve the quality and scale up of SEL implementation. Achieving systemic SEL requires an implementation support system that strategizes, coordinates, and tailors capacity-building strategies to local contexts [4]. Research demonstrates that well-aligned implementation support strategies (e.g., coaching) are associated with improved capacity for school-wide implementation and higher frequency of SEL practices in classrooms [11, 13, 16, 17]. However, the lack of infrastructure to support implementation prevents best practices from being scaled up [18]. This infrastructure needs to be responsive to local communities, as regions differ in their resources, needs, and capacities, and may require varied implementation support to be effective and sustainable [19].

There is the potential for broader components of educational systems, such as state departments of education and their regional delegates [20], to fill this infrastructure gap and support implementation. In the U.S., most states have developed SEL standards or provide SEL implementation guidance, whether mandated or voluntary, to support whole child development [21]. *How* local education agencies (LEA) then enact state-required or recommended policies or principles is highly variable. Specifically in California, the state has “SEL Guiding Principles,” and COEs act as a bridge between the state and districts to support local implementation of high-quality, systemic SEL at scale [22]. In the following section, we turn to central frameworks from implementation science to provide guiding concepts for this paper, which identify various potential forms of implementation support within multiple levels of the educational system to advance systemic SEL.

## Central implementation frameworks

We present two interconnected frameworks that examine the implementation of innovations (i.e., programs, policies, processes, and principles) that disseminate new knowledge for wide-scale implementation (p.172) [5]. The first framework is the *Interactive Systems Framework for Dissemination and Implementation* (ISF; [5]), which describes three systems that work in concert to implement innovations and ultimately support positive outcomes. The *synthesis and translation system* serves to translate research into application-ready practices that improve outcomes [5]. The *support system* builds the capacity of practitioners to implement research-based practices. Lastly, the *delivery system* consists of practitioners responsible for implementing research-based practices, such as teachers using new strategies to cultivate social and emotional skills in students.

The second implementation framework is the *Evidence-Based System for Implementation Support* (EBSIS; [6]), which expands on ISF by detailing *how* the support system builds the capacity of the delivery system via four implementation support strategies: training, technical assistance, tools, and quality assurance/quality improvement. Leeman and colleagues’ [23] systematic review further refined the EBSIS by adding two new implementation support strategies (i.e., incentives and peer networking) and by categorizing the implementation support by *orientation* (i.e., push, pull) in greater detail. Below, we elaborate on each of the original and expanded EBSIS components.

The first EBSIS implementation support strategy is *training,* which is planned information-sharing and skill-building (e.g., professional development; [6]). *Tools* are online or print informational resources (e.g., presentation slides, checklists) used to plan, educate, or evaluate skill building. *Technical Assistance* (TA) refers to targeted, individualized support [6], such as coaching and one-on-one consultations. *Quality Assurance/Quality Improvement* (QA/QI; [6]) strategies monitor quality and provide feedback for continuous improvement; QA/QI is also known as “assessment and feedback” [23], “feedback systems” [24], and “feedback loops” [19]. *Incentives* are financial and physical resources used to motivate capacity building [23]. *Peer networking* occurs when practitioners connect and learn from each other [23]. Additionally, Leeman and colleagues [23] advance the concept of *orientation* to describe the selection of interventions as either *push* or *pull*. A push orientation refers to the support system encouraging a set of predetermined interventions for the delivery system’s use. In contrast, a pull orientation describes how the system supports the implementation of interventions selected by the delivery systems based on their preferences [23].

These conceptual frameworks identify key systems, roles, and approaches to increase capacity, providing clarity to implementation researchers. However, there remains an opportunity to elaborate on these theories to increase the practical relevance of these frameworks for education leaders engaging in new initiatives. Below, we present contributions from extant work to highlight how implementation frameworks inform the scale-up of regional efforts.

## Empirical exploration of state-level support systems in implementation efforts

We reviewed the literature to identify studies applying ISF and EBSIS to regional, state, or national level (i.e., above the district) support systems seeking to scale-up efforts supporting well-being. Typically, these studies explored implementation support strategies as mutually exclusive, highlighting specific individual strategies. For example, prior work has focused on technical assistance (e.g., [25]; see [26] for a scoping review) or peer learning (e.g., [27]) with limited attention to other implementation supports. One study focused on technical assistance, which overlaps with other implementation supports [28], but does not consider the full range of EBSIS strategies and their combinations, which was identified as an important next step. These results offer valuable insights into real-world support strategies; however, the limited discussion of the potential support strategies that can be used in tandem limits the generalizability to systems using the full range of support strategies in an iterative and integrated manner.

One notable empirical study [29] investigated multiple forms of implementation support provided by a support system in tandem. The National Center for School Mental Health (NCSMH) is a federally funded training and technical assistance center that builds the capacity of school mental health providers through a national learning collaborative with school district representatives. The NCSMH provided training, TA, opportunities for peer learning, and tools for quality improvement and accountability. Researchers found that the key components for success included commitment from school and state-level participants, training for state leaders to provide TA, peer support, and continuous quality improvement [29]. Lastly, the researchers suggest that the “bundled delivery” of key learning collaborative components such as training, TA, and peer learning is what gave rise to systems change (p. 417) [29]. The current study responds to Bohnenkamp and colleagues’ call to examine “bundled” implementation supports. In examining how ISF and EBSIS apply to COEs providing implementation support, this study seeks to generate knowledge relevant to the practice of systemic SEL aided by integrated and responsive implementation support strategies.

## Overview of CalHOPE: implementation support in action

The present study focuses on CalHOPE Student Support (CalHOPE henceforth), an effort to advance systemic SEL statewide in California and support preK-12th grade educators as frontline responders for youth after the COVID-19 pandemic. In 2020, the Federal Emergency Management Agency (FEMA) and the Substance Use and Mental Health Services Administration (SAMHSA), via the California Department of Healthcare Services, funded the Sacramento County Office of Education in partnership with the Orange County Department of Education to launch a statewide system of SEL implementation support. A state planning team consisting of these practice-side leaders was joined by research-side leadership (University of California, Berkeley), including university-based research translation partners (Greater Good Science Center; [30]). The CalHOPE theory of action, *SHIFT-SEL*, advances social and emotional well-being through four systemic levers of transformation (i.e., partnerships, supports, capacities, structures and routines of SEL implementation) [19]. This model facilitates COEs to partner across the education system to provide tailored implementation supports and enhance regional capacity for improving the structures and routines of SEL delivery in districts and schools [20]. The current study focuses on the *supports* lever*,* which includes “funding, resources, training, coaching, and feedback systems” provided by COEs to districts and schools (p.5) [19].

Within CalHOPE, the multi-level partners align with ISF. Most relevant to the current study, COEs are regional *support systems* that bring state-level resources to their counties and support local educators to learn and deliver new SEL practices. Local district and school staff operate as the *delivery system*, receiving resources and implementing SEL in schools and classrooms (see [19] for additional details about the application of ISF to CalHOPE). Together, partners coordinate their expertise on practice, implementation, and research to provide a *nested system of support* [29] in which the state team supports COEs striving to build the SEL capacity of local educators and improve youth well-being across California.

In California, each of the 58 COEs plays an important role between state leadership and local districts and schools by administering funding and supporting local implementation of statewide initiatives. CalHOPE leverages COEs as regional hubs to facilitate *Communities of Practice* (CPs),^3^ which are the central implementation support strategy (i.e., training) to build local capacity for systemic SEL. Representatives from each COE participate in a monthly state-level CP on various topics (e.g., trauma-informed practices) facilitated by members of the state planning team to build relationships, problem-solve, disseminate resources, and improve practice [31]. Then, these state-level CP attendees host their own monthly county-level CPs tailored to regional needs to build the SEL knowledge and skills of local district and school-based educators. The specific content and facilitation of each COE’s CP, in addition to the other implementation support strategies they planned to enact to advance systemic SEL, vary county to county, but the monthly CPs were central to each region’s plan.

## Current study

As part of CalHOPE, COE leaders in California are committed to increasing SEL implementation support across the education system to bolster well-being in educators and students. The COE leaders articulated their county’s actions and commitments for CalHOPE participation and SEL implementation via written plans in Spring 2021. The current study engages in a qualitative analysis of these implementation plans. Guided by ISF, EBSIS, and their recent refinements, we answer the following research question: How do COE leaders leverage implementation support strategies to build their local districts’ capacity to advance systemic SEL efforts? By describing how and in what ways implementation support strategies may advance systemic SEL, we fill a gap in the research literature by taking a comprehensive approach to studying the provision of integrated and responsive implementation support strategies in practice.

## Method

### Data and procedures

A total of 97% (56 of 58) of California counties signed Memoranda of Understanding (MOUs) to participate in CalHOPE. Each County Superintendent of Schools appointed “County Leads”, a team of two to four COE representatives, who began meeting with the CalHOPE state planning team in January 2021 [32]. The County Leads developed and submitted implementation plans to the state planning team by March 2021 as required by the MOU terms; these plans detailed how they intended to provide ongoing implementation support between January and June 2021 for systemic SEL in their respective counties. Specifically, County Leads responded to four open-ended questions to describe the following: 1) activities related to the planning, development, and implementation of their County CP,^4^ including the county team composition; 2) ways to recruit a wide set of educators and nonprofits to participate in the CP; 3) technical assistance for educators and nonprofits to implement SEL within the CP; and 4) a descriptive timeline. The state planning team used the implementation plans to support counties through progress monitoring and technical assistance.

Plans ranged from 524 to 2001 words. Per a series of MOUs, counties consented to having materials submitted for reporting purposes be de-identified (i.e., replaced names of people, places, and organizations with numerical codes to improve confidentiality), and stored in a research archive for subsequent analysis; research protocols were approved by the University of California, Berkeley’s Office for Protection of Human Subjects (OPHS). This study analyzes all 56 plans articulating how counties intended to facilitate monthly County CPs for district leaders, school staff, and youth-serving organizations, and provide SEL implementation support from March-August 2021.

### Qualitative analysis

The first three authors led the qualitative analysis; we have direct experiences working in U.S. public schools as a teacher, school social worker, and youth leadership consultant, are racially, ethnically, and linguistically diverse, and have direct experiences living, working, and being students in California. We analyzed the county SEL implementation plans using directed qualitative content analysis (QCA; [33]). Directed QCA is a strategy in which pre-existing frameworks are applied deductively to systematically identify patterns in textual data, while allowing for an iterative, inductive approach to revise codes, generate themes, and extend the original frameworks. We engaged in this coding process until the themes were substantive, consistent, and explanatory, and no new concepts were identified. This study is in accordance with the Standards for Reporting Qualitative Research (SRQR) guidelines.

We employed direct QCA in three phases (i.e., preparation, organization, and reporting; [34]) using the steps appropriate for analyzing qualitative practice-generated policy documents. In the *preparation* phase, we refined skills in qualitative coding and analysis, prepared the implementation plans for secondary analysis and coding, and began the data immersion process [33]. The *organization* phase included developing the main coding categories based on prior frameworks, determining coding rules, testing the preliminary codebook, and applying and analyzing codes. We then completed the *reporting* phase by documenting our analysis and sharing thematic findings with quantitative summaries and direct examples in this paper’s results section.

#### Coding procedures and refinements

The researchers met weekly to develop a preliminary codebook, approach coding consensus, and discuss thematic analysis. Codes were deductively drawn from the implementation support strategies identified in the original EBSIS framework (i.e., training, tools, technical assistance, feedback loops) and more recent elaborations (i.e., incentives, peer networking; [23]). The coding protocol clarified the application of codes only to explicitly stated implementation strategies (e.g., the SEL team is conducting a teacher survey to collect PD feedback) and not to speculative, general, or vague descriptions (e.g., we plan to create surveys). Preliminary codes were piloted in a subset of six plans. To improve coding consistency, all three researchers coded plans independently before meeting to compare and discuss the coding process; then, the codebook was refined via consensus, and examples were added. Refinements included adding two subcodes to *training* to better understand how counties planned different types of learning: *directed learning*, which is didactic and instruction-oriented, and *peer learning,* which leverages interaction and co-learning among participants (inspired by *peer networking* as proposed in Leeman et al., 2015 [23] and specifically applied to training). Additionally, we refined codes for *orientation,* which have been used previously [23] to describe the support system *pushing* specific interventions out to the delivery system or the delivery system *pulling* interventions from a menu of options. To better align with CalHOPE’s goals and also reflect COEs’ agency in writing the implementation plans, we broaden *orientation* from its original intervention-focused definition to also examine the support strategies themselves. In our conceptualization, *pushing* entails the COE support system using a “top-down” approach to both select and determine the implementation support strategies. Conversely, *pulling* entails a “bottom-up” approach accounting for the delivery system’s perspectives and preferences, such that the capacities of both the support and delivery systems inform support strategy selection.

Two researchers conducted the main coding by independently applying the finalized codebook using Dedoose (version 9.0.1.7) [35]. To establish coding consensus, one member of the team coded the entirety of all plans, and another coded one of four sections within all plans. The three coders met weekly to discuss discrepancies in double-coded sections; the first author served as a tie-breaker to resolve more challenging discrepancies. A consensus-based approach allowed researchers to leverage their multiple perspectives and engage deeply with each plan to make meaning of the complex and varied data [36, 37]. After reaching consensus, the first three authors met regularly to interpret the data, discuss patterns and notable cases related to the research questions, generate preliminary themes, and finalize the themes in collaboration with all authors.

## Results

This qualitative document analysis, informed by the ISF [5] and the EBSIS [6, 23] sheds light on how COE leaders articulated their commitment to provide implementation support to enhance SEL in the aftermath of the COVID pandemic. We found that across the implementation plans, the EBSIS strategies (i.e., trainings, tools, technical assistance, feedback loops, incentives) were referenced. In addition, our thematic analysis contributes to the literature by offering new ways to select and offer implementation support strategies. First, we present how COE leaders planned to creatively use the five implementation support strategies independently and *in combination* to advance systemic SEL implementation in integrated and responsive ways (e.g., embedding the release of SEL *tools* within monthly Community of Practice *trainings*). Second, as a result of our inductive analysis, we put forth an innovative conceptualization of implementation support strategies by visioning how strategies fall along two dimensions, *implementation support strategy orientation* (inspired and adapted from Leeman et al., [23]) and *implementation support strategy facilitation* (inductively developed)*,* which we refer to as *support orientation* and *support facilitation* henceforth, to describe how these support strategies reflect current need and capacity.

### Overview of implementation support strategies in action

Within implementation plans, COE leaders enumerated various approaches aligned with the five main EBSIS strategies (see Table 1 for definition, operationalization [38], and example for each strategy; see [32] for more information about CalHOPE implementation partners/actors). For each implementation support strategy, we provide examples and report the proportion of plans using the strategies to describe the overall frequencies.

**Table 1:** Definition, Operationalization, and Example for Each Implementation Support Strategy

Implementation Support Strategy	Definition & Operationalization	Example
Training	Planned information-sharing and skill-building sessions (e.g., professional development) often conducted in group settings [6]. May be delivered by county leaders, local educators, or consultants with the goal of increasing the knowledge of CP attendees.	*“Each SEL CoP will follow the statewide SEL CP and use the materials offered to the [Redacted] COEs. Additionally, we will include district and school spotlights as well as networking and planning time within each SEL CP. Our goal is to include district and school teachers and leaders as much as possible and respond to their immediate needs…”*
Tools	Informational resources that share knowledge or help users organize knowledge [6]. These resources (e.g., manuals, SEL frameworks) are curated or developed by COE leaders to advance the SEL learning goals of local practitioners.	*“The Climate Coach produced videos for the [CP], which explicitly teach SEL strategies. The videos will become part of a hub of SEL resources for teachers to use in their classrooms that will be linked to the [Redacted] COE webpage.”*
Technical Assistance	Individualized support to build an organization's capacity to implement an innovation [6]. Often COE leaders or consultants provide targeted skill-building support (e.g., coaching, site visits) to local practitioners with varying frequency.	*"Additionally, [Redacted Individual], Coordinator of the [Redacted County COE] SEL CP is offering office hours twice a month on Thursdays from 4–5 pm, and will be hosting a series of free guided SEL systems planning sessions for district teams."*
Feedback Loops	Strategies that provide feedback to practitioners for reflection and continuous improvement [6, 19]. These progress monitoring approaches (e.g., benchmark trackers, checklists) can be completed by COE leaders or local practitioners to identify ways to learn and improve.	“*Connecting [Local Education Agencies] to and supporting districts in the administration of self-assessment tools that sites can use to determine their own level of school-wide SEL implementation.*”
Incentives	Providing financial and physical resources to support capacity-building efforts [22]. These resources are provided to local educators as a result of participation in specific aspects of CalHOPE as determined by COE leaders.	*“We have 32 districts in County [Redacted] and are offering stipends for two (2) participants from each district to participate in the CPs, complete a district SEL self-assessment, and draft a small project with the goal of improving site-based SEL.“*

All county plans (*N* = 56, 100%) mentioned *training* as an implementation support strategy they plan to use (e.g., CP, professional development). These trainings took multiple forms, with combinations of didactic, lecture-based, and peer learning elements. *Tools* included online and physical resources like SEL curricula, books, and videos, and were the second most mentioned support strategy (*n* = 54, 96%). The vast majority of plans (*n* = 48, 86%) included *technical assistance*, with examples including providing office hours, site visits, and coaching. *Feedback Loops*, such as SEL screening tools and tracking implementation progress, were the least commonly mentioned implementation support strategy (*n* = 15, 27%). Nearly half (45%; *n* = 25) of the COEs mentioned using *incentives* (e.g., stipends, mini-grants) to motivate participation and knowledge sharing.

### Simultaneously integrating implementation support strategies

In addition to using discrete or isolated implementation support strategies to advance SEL capacity building, we noted how COE leaders creatively mentioned combinations of these five strategies (i.e., bundling; [23, 39]) to strengthen implementation efforts. In this section, we present three commonly referenced combinations of implementation support strategies in the plans: 1) Tools + Training; 2) Training + Technical Assistance; and 3) Incentives + any of the four original EBSIS strategies.

#### Combination 1: Tools + Training

Counties often used training, including CPs, as a space for resource (i.e., tool) brokering. This included distribution of state-level turnkey resources and peer-to-peer sharing of tools. One COE described how they “would hold quarterly large SEL Community of Practice meetings which [would] utilize the Greater Good Science Center modules and include space for networking and sharing of effective tips and ideas.” Another county described how their CP would bring district teams together to “share best practices and build collective capacity” with the goal of “[providing] district teams the opportunity to create, vet, and share high-quality resources and tools.”

#### Combination 2: Training + Technical Assistance

In the plans, training was often bundled with technical assistance to supplement and deepen newly disseminated knowledge. For example, one county reported that they would host a training on Adverse Childhood Experiences and Trauma-Informed Practices with technical assistance, “…including follow-up support for them so that they can best serve our students.” Another county similarly noted that they would host “monthly SEL CPs for the County Team as a follow up to what was learned during the multi-county SEL CP training”; they aim to support the “retention of the information provided, reinforce key concepts discussed in the initial training, and allow for the COE to provide targeted technical assistance in requested areas by our team.”

#### Combination 3: Incentives + Other Strategies

Incentives were commonly bundled with several other implementation support strategies to motivate, enable, or reward participation in implementation strategies [23]. Counties had access to flexible state-allocated funds, and many counties used financial incentives for educators to participate in SEL trainings and other school activities (e.g., implementing curriculum/tools). One county wrote that participation in the CP (i.e., training) would “be incentivized with a $1000 stipend for each participant attending all meetings as well as helping to develop a plan for implementation of SEL in their district through our CP.” Another county similarly “offered stipends for teachers to attend all SEL CPs [training], complete the first module of GGSC [tool], and implement 4 strategies in their classroom [tool].”

### Conceptualizing the dimensions of support orientation and support facilitation

The research team made coding decisions to indicate whether an implementation support strategy was present or absent, but also noticed patterns and made comparisons among coded text. Thus, beyond treating these codes in a binary fashion, we inductively developed a new conceptualization that systematically organized support strategies along two intersecting dimensions. The first dimension, *support orientation*, expands the notion of *orientation* from prior work, which specifically focused on interventions [23], to describe the degree to which implementation support strategies were conceptualized to be more pre-determined by County Leaders (pushed, top-down) or more driven by local preferences and interests (pulled, bottom-up). The second dimension is *support facilitation*, which emerged during the analysis process to capture the support strategy’s level of requisite preparation (i.e., less or more preparation) in its planning and execution. Next, we describe these two dimensions and present examples of implementation support strategies mapped along these dimensions.

#### Support orientation: top-down and bottom-up approaches to implementation support

**Fig. 1:**
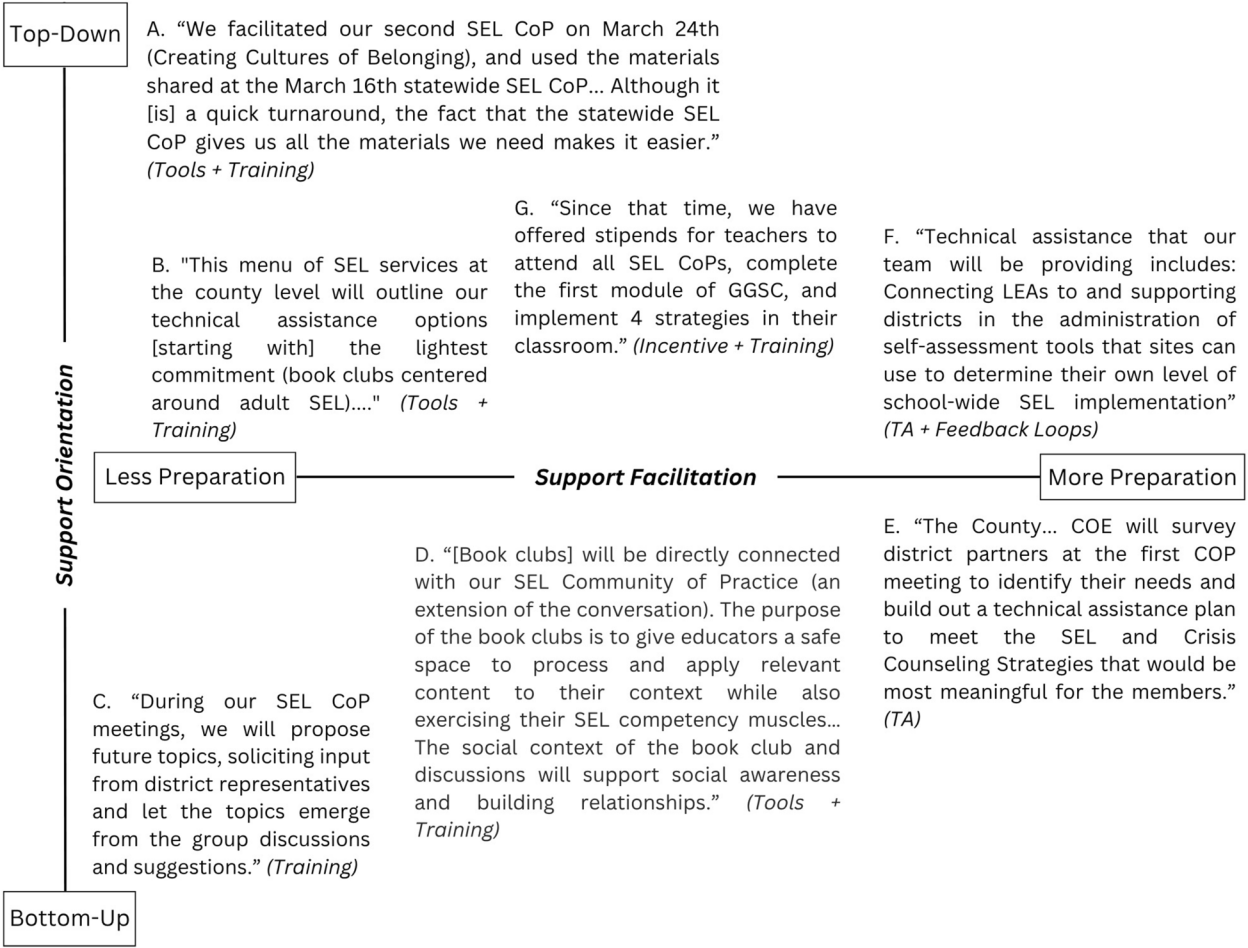
Illustration of Examples along the Dimensions of Support Orientation and Support Facilitation

The first dimension (Fig. 1, y-axis) reflects the *support orientation* of an implementation support strategy, ranging from *top-down* to *bottom-up*. Strategies with a greater top-down orientation are more didactic, prescribed knowledge transfers; this is akin to how Leeman and colleagues [23] define “push.” Using training as an example, strategies coded as “directed learning” align with this top-down orientation, given its predetermined training content. In comparison, “peer learning” is a bottom-up oriented strategy, characterized by practitioners learning from each other in horizontal knowledge exchanges via discussion, networking, or crowdsourcing ideas (i.e., “pull”; [23]). To illustrate this, we turn to Example A in Fig. 1 to depict an instance of a combination of Tools+ Training as an implementation support strategy with a high top-down orientation:

We facilitated our second SEL CP on March 24th (Creating Cultures of Belonging), and used the materials shared at the March 16th statewide SEL CP... Although it [is] a quick turnaround, the fact that the statewide SEL CP gives us all the materials we need makes it easier.

In this approach, the state-level team provided agendas and activities in a practice-friendly format to facilitate their uptake in regional CPs. Then the County in Example A is taking already developed trainings and directly sharing them with their local CPs. The content is pre-determined with no adjustments or incorporation of local perspectives, which is why the orientation is high in its top-down approach.

Other efforts can be more bottom-up and pull in local needs and interests. This may involve distributing SEL resources developed or recommended by local practitioners or having breakout groups or networking opportunities during CP meetings for attendees to share specific successes with SEL activities. Example D on Fig. 1 is an instance of an implementation support bundle (Tools + Training) with a high bottom-up orientation:

[Book clubs] will be directly connected with our SEL Community of Practice (an extension of the conversation). The purpose of the book clubs is to give educators a safe space to process and apply relevant content to their context while also exercising their SEL competency muscles…The social context of the book club and discussions will support social awareness and building relationships.

Here, the COE leaders planned to facilitate their CP as a book club; however, the specific topics are not predetermined by County Leaders and will be determined by the local attendees, which is a bottom-up approach.

#### Support facilitation: implementation support strategies ranging in level of requisite preparation

The second dimension, *support facilitation*, was inductively identified in the analysis process and captures how support strategies ranged from requiring less preparation (e.g., less structured or burdensome in planning or easier to facilitate) to more preparation (e.g., more structured or requiring greater levels of planning or skill to facilitate) in its facilitation (Fig. 1, x-axis). On Fig. 1, Example F is a bundle of TA+ Feedback Loops that represents a relatively high degree of preparation by the County: “Technical assistance that our team will be providing includes: Connecting LEAs to and supporting districts in the administration of self-assessment tools that sites can use to determine their own level of school-wide SEL implementation.” This particular form of SEL implementation support requires the development, completion, and analysis of self-assessment tools to guide reflection about local SEL practices; this is a highly involved and iterative process requiring intensive planning and advance preparation by the support system. In contrast, Example C in Fig. 1 describes a training approach involving less preparation in its facilitation: “During our SEL CP meetings, we will propose future topics, soliciting input from district representatives and let the topics emerge from the group discussions and suggestions.” Here, COE leaders are creating opportunities to obtain feedback from attendees in a less intensive way during regional CP meetings to discover important and salient topics.

## Discussion

Guided by two implementation science frameworks (ISF and EBSIS), the current study’s directed qualitative content analysis of implementation support plans provides insight into how support systems in California are building the capacity of local educators to implement systemic SEL. Scholars have put forth a call to action regarding the need to systematically investigate implementation support (e.g., [12]), and this study addresses this gap and contributes insights to both research and practice. The implementation support documents drafted by COE leaders reveal how counties plan to leverage individual implementation support strategies and also use combinations of strategies. Additionally, we identified two dimensions of implementation support that may aid education leaders when engaging in early implementation efforts and decision-making processes. First, counties varied in the extent to which they held a top-down or bottom-up orientation when selecting implementation support strategies, which may reflect responsiveness to the local context’s preferences, skills, or needs. Second, implementation support strategies ranged in the degree of advance preparation required for their facilitation, which signals the importance of estimating both the support and delivery systems’ capacity when engaging in decision-making to ensure the feasibility of implementation plans. We explore these themes in conversation with the broader literature and future directions below.

Recently, Wandersman and colleagues [40] put forth ISF 2.0, which includes enhancements on how practice models can drive research, especially given the important role of societal factors, funding, and community need. Our study provides a concrete illustration of how community-driven implementation support strategies can be feasibly scaled up, delivered in education settings, and be responsive to culture and climate. We found the following: the majority of plans mentioned the use of training (which was expected, as CPs were part of CalHOPE’s design and MOUs), tools, and TA, half the plans mentioned incentives, and a quarter mentioned feedback loops. In addition to using individual strategies, County Leaders creatively bundled implementation support strategies, which have been documented in prior empirical work. For instance, Gayles and colleagues [28] noted how one practice can map onto several EBSIS strategies; they stated that systematic investigation of these combinations was beyond their scope, but worthy of further exploration. Thus, the current study builds on this work by exploring the interconnected nature of support strategies and uncovering three common bundles, which can be adopted or replicated in other jurisdictions or initiatives: Tools + Training, Training + TA, and Incentives + another EBSIS strategy. Integrated support strategies may be efficient, mutually reinforce each other, and ultimately create a stronger support system [6]. Future work can examine whether combinations of support strategies have a greater effect on the delivery system’s engagement, capacity, and implementation routines, which can then advance a deeper understanding of the process of implementation and inform future capacity-building efforts.

We found that implementation support strategies can be conceptualized via two intersecting dimensions, support orientation and support facilitation, which capture aspects of the support and delivery systems’ responsiveness and capacity. Support orientation expresses how a support strategy may range on a continuum of being top-down or bottom-up, which reflects the degree of responsiveness. Previous research has noted a continuum of TA ranging from being more content-driven (i.e., top-down), which is the transfer of predetermined knowledge, to more relationship-based (i.e., bottom-up), which includes individualized and co-created learning [41]. Thus, support orientation reflects the importance of the alignment between need and support [25]. This finding aligns with emerging work on multilevel adaptive implementation strategies (MAISYs; [42]) by providing examples of implementation support decisions made by leaders at the county-level to meet specific contextual needs within the education system. Future work can empirically examine decision-making by support system providers to understand their role in implementation support, which may differ based on whether they are in the early, middle, or late stages of implementation, as related to outputs and outcomes.

The second dimension, support facilitation, captures how implementation support strategies require different levels of advance preparation and skill, which has been found in prior research exploring formality (e.g., [43, 44]) and flexibility (e.g., [45]). Our data are from the start of CalHOPE implementation, and understanding the realistic capacities of the support and delivery systems was critical to decision-making and resource allocation. It may be particularly useful for support systems to consider the degree of planning and bandwidth required to carry out these strategies. For instance, strategies that require less preparation may be less demanding initially [23]; then, strategies involving more planning and skill in facilitation become increasingly appropriate and feasible over time as the systems increase their capacities. These contextual factors that influence the implementation process [4] and the relationship between the support and delivery systems [46] are critical to effective implementation planning.

### Limitations and future directions

This study examines initiated and planned implementation activities; the analysis of actual ongoing implementation supports, subsequent implementation quality, and associated outcomes are outside the scope of the current study. We encourage future work to examine planned implementation through a longitudinal lens, associating planned and completed implementation support strategies – both individually and in bundles – with implementation outputs and population-level outcomes; this aligns with ISF 2.0’s elaboration on measuring implementation outcomes and individual/community outcomes separately [40]. In addition, some implementation support strategies or bundles may be more effective or drive particular outcomes; future work assessing goal articulation and motivations alongside implementation mechanisms and outcomes in context [47] would be beneficial, especially in resource-limited environments. Additionally, our analysis focuses on 56 counties with substantial heterogeneity, but they are all from one state, which may limit transportability. Lastly, several regions are undergoing impressive and innovative implementation efforts to bring innovations into educational settings; however, the research base lags behind. There is a need for additional systematic and rigorous research to capture support system activities in real-time and evaluate comprehensive infrastructure-building efforts that are critical for the everyday experiences of youth and educators.

## Data Availability

Additional details about the data and materials can be obtained by emailing the first author.

## Footnotes

1 We specifically use the language of “implementation support strategy”, rather than implementation strategy, to describe implementation strategies provided by the support system.

2 The 58 counties in California partner with the state Department of Education to support nearly 10,000 public schools in over 1,000 school districts (California Department of Education, 2024).

3 Local districts have abbreviated Community of Practice as either CP or CoP. In this paper, we use CP and have updated all instances when CoP were originally mentioned to CP to maintain consistency.

4 County-level SEL Communities of Practice.
